# An Antifungal Role of Hydrogen Sulfide on *Botryosphaeria Dothidea* and Amino Acid Metabolism Involved in Disease Resistance Induced in Postharvest Kiwifruit

**DOI:** 10.3389/fpls.2022.888647

**Published:** 2022-06-16

**Authors:** Bing Duan, Huaying Du, Wei Zhang, Jing Wang, Zhipeng Cai, Yonggen Shen, Tenghuan Huang, Jie Yuan, Zengyu Gan, Jinyin Chen, Liqin Zhu

**Affiliations:** ^1^College of Food Science and Technology, Jiangxi Agricultural University, Nanchang, China; ^2^Jiangxi Key Laboratory for Postharvest Technology and Nondestructive Testing of Fruits and Vegetables, Collaborative Innovation Center of Postharvest Key Technology and Quality Safety of Fruits and Vegetables, College of Agronomy, Jiangxi Agricultural University, Nanchang, China; ^3^College of Materials and Chemical Engineering, Pingxiang University, Pingxiang, China

**Keywords:** hydrogen sulfide, kiwifruit, *Botryosphaeria dothidea*, disease resistance, amino acid metabolism

## Abstract

*Botryosphaeria dothidea* is a major pathogen responsible for postharvest kiwifruit soft rot. This study aimed to determine the influence of hydrogen sulfide (H_2_S) on postharvest resistance to kiwifruit soft rot and the antifungal role of H_2_S against *B. dothidea*. The results indicated that H_2_S (20 μl L^−1^) restricted the lesion area following inoculation with *B. dothidea*. H_2_S enhanced the production of shikimic acid, tyrosine, tryptophan, and phenylalanine while also increasing the total phenols, flavonoids, and lignin. H_2_S upregulated the expression of *AcDHQS, AcSDH, AcSK, AcPAL, AcCAD*, and *AcCHS*. Additionally, sodium hydrosulfide (NaHS)-released H_2_S inhibited mycelial growth. NaHS concentrations of 20 and 40 mmol L^−1^ significantly decreased the mycelial weight and malondialdehyde content (MDA) content while increasing cell membrane conductivity and membrane leakage. The results indicate that H_2_S induces resistance in kiwifruit *via* a microbicidal role and amino acid metabolism involved in postharvest kiwifruit disease resistance.

## Introduction

Kiwifruit is well-known as “the king of fruit” because of its refreshing taste and rich VC content, making it extremely popular. However, as a typical respiratory climacteric fruit, kiwifruit may be impacted and squeezed during transportation and is easily softened and even rots after being infected with fungi (Prencipe et al., 2016; Kai et al., 2020). These physiological phenomena impede the development of the kiwifruit industry. *Botryosphaeria dothidea* is one of the main pathogens responsible for kiwifruit soft rot (Zhang et al., 2020). Therefore, it is particularly important to study a safe and effective postharvest method of kiwifruit (Dai et al., In Press). It is considered one of the most promising methods for controlling fruit postharvest diseases by inducing resistance to activate the fruit defense system. Recently, exogenous substances such as chitosan, methyl jasmonate, and nitric oxide have been used to induce postharvest disease resistance of fruit (Pan et al., 2019; Rajestary et al., 2021; Yang et al., 2021).

In plants, hydrogen sulfide (H_2_S) participates in signal transduction and regulates development, maturation, senescence, and other physiological processes. H_2_S activates disease resistance to pathogens and improves fruit resistance to postharvest stress. Exogenous H_2_S application has become an anticipative approach for fruit quality improvement. In our previous studies, H_2_S could extend the shelf life of fruit by improving antioxidant activity, modulating phenolic metabolism, and inhibiting browning (Zhu et al., 2014; Sun et al., 2015; Dou et al., 2021). Additionally, the transcriptome sequencing analysis proved that H_2_S was intimately associated with kiwifruit cell wall degradation and retarded kiwifruit softening (Lin et al., 2020). A previous study revealed that treating fresh-cut sweet potatoes with 2 mmol L^−1^ NaHS effectively inhibited the black rot and soft rot of fruit and palliated the rot degree of sweet potatoes (Tang et al., 2014). H_2_S treatment restricted spore germination and mycelial growth of *Aspergillus niger* and *Penicillium italicum* (Fu et al., 2014). Hu et al. (2014) demonstrated that H_2_S significantly inhibited the spore growth of *A. niger* and *P. italicum* in fresh-cut pears and that H_2_S could better maintain the quality of pears during storage.

During long-term coevolution of plants and pathogens, plants develop a series of disease-resistant responses to pathogen infection. Hosts produce disease-resistant substances by regulating their physiological metabolism to resist pathogen invasion and prevent or delay the onset of diseases, while the synthesis of disease-resistant substances is intimately linked to amino acid metabolism and secondary metabolism of hosts (Zeier, 2013; Jan et al., 2021). As necessary nutrients for plant growth and development, certain amino acids have sanguine repercussions in resisting pathogen infection (Kadotani et al., 2016; Canfield and Bradshaw, 2019). Although previous research has illustrated that plants are usually accompanied by amino acid accumulation under abiotic or biological stress (Yun et al., 2015), there are still relatively few studies on amino acid metabolism in fruit postharvest diseases. Kumar et al. (2020) discovered that phenylalanine application before or after harvest inhibited the suppression of pathogen-induced postharvest decay. Sun et al. (2019) revealed that disease resistance of tomato fruit could be improved by inducing glutamate receptor expression and promoting amino acid accumulation. In another study, exogenous glutamic acid treatment induced pear fruit resistance to *Penicillium expansum* by activating defense-related protein and amino acid metabolism (Jin et al., 2019). Differentially expressed proteins (DEPs) were significantly enriched in the amino acid metabolism pathway; salicylic acid (SA), oligochitosan, and *Pichia membranaefaciens* reduced resistance to *Geotrichum candidum* in citrus fruit using protein omics (Wang et al., 2020b).

This study sheds light on the inhibitory effect of H_2_S treatment on kiwifruit soft rot caused by *B. dothidea* and its possible defense mechanism, including *in vitro* antifungal activity, defense-related gene expression, amino acid metabolism, and secondary metabolites.

## Materials and Methods

### Plant Material and H_2_S Treatment

Kiwifruit (*Actinidia chinensis Planch*. cv. Jinyan) was picked from an orchard in Fengxin County, Jiangxi Province, China (longitude: 115.4° E, latitude: 28.7° N). The fruit was delivered to the laboratory at once upon harvest. Kiwifruit was uniform in terms of shape, size, color, and absence of visual defects for subsequent experiments. The selected kiwifruit was randomly divided into two groups. H_2_S-treated (H_2_S) group was fumigated with 20 μl L^−1^ of H_2_S gas (99.99% purity) at 20°C for 30 min (Lin et al., 2020), whereas the control (CK) group was not treated. Each treatment consisted of 150 fruit with three replicates.

### Pathogen Inoculation and Sample Collection

*Botryosphaeria dothidea* was isolated and purified from infected kiwifruit and cultivated in potato dextrose agar medium (PDA) at 25°C for 1 week. After 24 h of H_2_S treatment, sterile tips were used to form a well-proportioned wound (3 × 3 mm) at the equator of each fruit. Following that, the spore suspension (10^6^ cfu ml^−1^) was prepared using sterile water. Kiwifruit was pierced at two opposite points in the middle, and 10 μl of spore suspension was injected into each wound.

The kiwifruit pulp sample was collected from the fruit's equator area at 0, 2, 4, 6, 8, and 10 days, frozen in liquid nitrogen, and stored at −80°C for the following assay.

### Lesion Area Determination on Kiwifruit

The lesion area was obtained by calculating the approximate circular area of lesion diameter. Lesion diameter on kiwifruit was measured using a vernier caliper.

### *In vitro* Antifungal Activity

The agar dilution culture was employed to observe the effect of H_2_S donor, NaHS, on the mycelial growth of *B. dothidea* as reported by He et al. (2018). NaHS concentration was set by the double and half dilution method. The potato glucose solid medium (i.e., PDA) was sterilized and cooled to ~60°C, before adding NaHS to achieve the final concentrations of 0, 1.25, 2.5, 5, 10, 20, 40, and 80 mmol L^−1^. After plate solidification, inoculation and culture were performed using the plate streaking method, and the mycelium growth was observed. After 7 days of culture, a bacteria cake (*d* = 7 mm) was formed on the medium with uniform growth, and each plate was inoculated with one bacteria cake. The bacteria cakes were cultured at 25°C for 7 days. The minimum inhibitory concentration (MIC) was the lowest concentration without obvious mycelial growth observed by naked eyes after 7 days. The diameter of mycelial growth circle was measured daily by the cross method. Each concentration was repeated three times.

### Mycelium Weight, Extracellular Conductivity, Membrane Leakage, and Malondialdehyde Content

The Erlenmeyer flask contained a 99 ml potato glucose liquid medium (i.e., PDB) and 1 ml spore suspension (10^6^ cfu ml^−1^). After shaking the culture for 5 days (28°C, 180 rpm), MIC and 2MIC NaHS were added to the culture solution, respectively. Then, after sequential shaking culture for 3 days, the hyphae were filtered with filter paper, washed three times with sterile water, and dried at 80°C. Each treatment was repeated three times. Hyphae were weighed using an electronic balance.

Liu et al. (2019) described the extracellular conductivity and leakage of the cell membrane. After inoculating 1 ml of spore suspension into 99 ml of PDB for shaking culture at 28°C, 180 rpm for 4 days, the mycelium was washed and centrifuged (4,000 *g*, 15 min). The precipitated mycelium was evenly mixed in phosphate buffer solution (PBS, pH 7.0). NaHS with MIC and 2MIC were treated for 0, 1, 2, 3, and 4 h, respectively. The influence of H_2_S on the extracellular conductivity of *B*. *dothidea* was determined using a micro conductometer, and the supernatant was gathered following centrifugation (10,000 *g*, 15 min). To determine cell membrane leakage, the supernatant was gathered after centrifugation (12,000 *g*, 2 min). The absorbance value was measured at 260 nm, and each concentration was repeated three times. The CK group was adjusted with PBS (pH 7.0).

Malondialdehyde (MDA) content was determined as described by Liu et al. (2021). In an ice-bath mortar, 1.0 g of hyphae and 5 ml of PBS (0.1 mol L^−1^, pH 7.0) were ground, with a small amount of quartz sand. The supernatant was gathered after centrifugation (10,000 *g*, 10 min). The mixed solution was composed of 2 ml of 0.5% thiobarbituric acid solution and 2 ml supernatant and incubated at 95°C for 20 min, followed by an ice bath for 5 min and centrifugated for 10 min (4°C, 12,000 *g*). We determined the absorbance values at 532, 600, and 450 nm. Each concentration was repeated three times. The MDA content was expressed as μmol kg^−1^.

### Real-Time Quantitative PCR

Kiwifruit pulp tissue was ground to powder with liquid nitrogen. Total RNA was extracted from kiwifruit samples using a quick RNA isolation Kit (Huayueyang, China). Using 4 × DNA wiper mix and 5 × Hirscript III QRT Supermix (Vazyme Bio, China) reduces RNA sample into cDNA, and the operation steps were conducted according to kit's instructions. The relative expression levels of genes were calculated using the 2^(−ΔΔCt)^ method (Pan et al., 2019), and each sample was performed for three technical replications. The primer sequences are listed in Supplementary Table 1.

### Determination of Related Amino Acid Metabolites

High-performance liquid chromatography (HPLC) was used to quantitatively analyze amino acid metabolites in kiwifruit tissue. The extraction and chromatographic conditions of amino acids were as follows, as described by Ge et al. (2019a).

Shikimic acid: 0.5 g kiwifruit pulp coupled with 3 ml 1.0 mol L^−1^ H_3_PO_4_ solution was mixed evenly, followed by ultrasonic extraction at 25°C for 30 min. Following centrifugation (25°C, 12,000 *g*) for 15 min, the supernatant was taken to analyze the shikimic acid content. The detection wavelength was 215 nm; the column temperature was 25°C; the mobile phase was 1 mol L^−1^ H_3_PO_4_ (HPLC grade); the injection volume was 10 μl; and the flow rate was 0.8 ml min^−1^.

Tyrosine, tryptophan, and phenylalanine acid: 0.5 g of kiwifruit pulp powder was mixed with 3 ml 0.05 mol L^−1^ KH_2_PO_4_ solution and extracted at 4°C for 24 h. Following centrifugation (12,000 *g*, 15 min) at 4°C, the residue was washed using 0.05 mol L^−1^ KH_2_PO_4_. The leaching solution coupled with washing solution was fixed to 10 ml with the mobile phase solution to analyze the three amino acids. The detection wavelength was 215 nm; the column temperature was 25°C; the mobile phase was 0.05 mol L^−1^ KH_2_PO_4_ methanol (v/v: 9:1); the injection volume was 15 μl; and the flow rate was 0.6 ml min^−1^.

All supernatants were filtered through a 0.22-μm microporous filter and used for subsequent liquid phase experiments. The quantitative analysis of all amino acids was performed on a waters C18 column (4.6 mm × 250 mm, 5 μm) HPLC (Agilent 1260). The standard curves were constructed using shikimic acid, tyrosine, tryptophan, and phenylalanine standards (Sorebol, Chromatographic pure). The contents of metabolites were expressed on the basis of fresh weight as mg kg^−1^.

### Determination of Disease-Resistant-Related Substances

Flavonoids and total phenolics content were determined as described by Zhu et al. (2016), with minor modifications. Kiwifruit powder (2.0 g) was mixed with 6 ml of 1% (v/v) HCl-methyl alcohol. The extract was centrifuged (10,000 *g*, 10 min) under 4°C to collect the supernatant. The content of flavonoids and total phenolics was expressed as g kg^−1^, with detection wavelengths of 280 and 325 nm, respectively.

Lignin content was determined as described by Liu et al. (2014), with minor modifications. Kiwifruit pulp powder (1.0 g) was mixed with 5 ml of 95% ethanol and centrifuged for 10 min to obtain a precipitate, which was washed with washing solution, followed by drying in an oven. The dried sample was mixed with 3 ml 25% glacial acetic acid bromide solution. After 30 min of incubation at 70°C, the reaction was terminated with 0.9 ml 2 mol L^−1^ NaOH. The sample was diluted to 10 ml with glacial acetic acid and centrifuged (4,000 *g*, 10 min). The absorbance value of the sample solution was determined at 280 nm. The lignin content was expressed as OD_280_ FW.

### Statistical Analysis

The data used the SPSS software version 22.0 for the analysis of variance. The data were expressed as mean ± SD (standard deviation), and the means were compared using the Duncan's multiple range test (*P* < 0.05). Figures were drawn using the Origin 2019 software.

## Results

### The Lesion Area of Kiwifruit After Inoculation With *B. dothidea*

**Figure 1:**
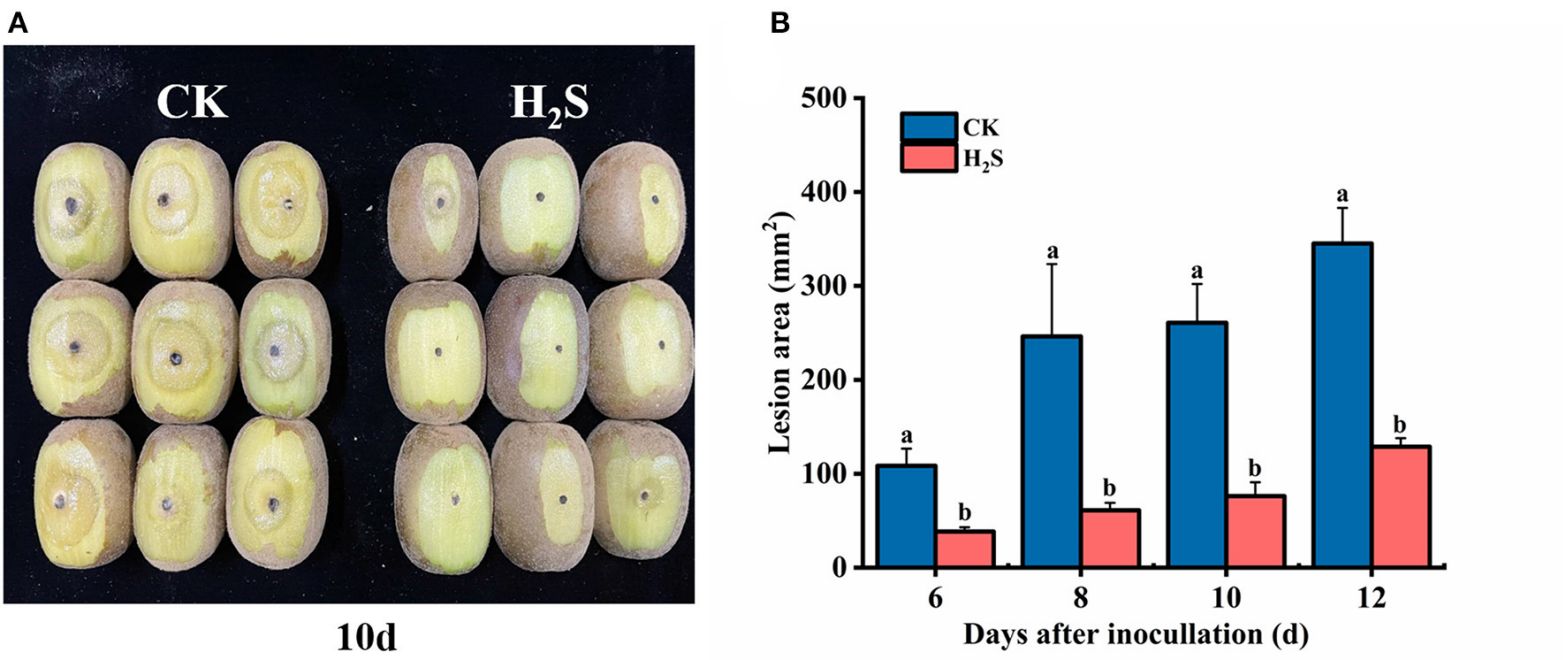
Infection symptoms **(A)** in kiwifruit at 10 days after inoculation with *Botryosphaeria dothidea*. The lesion area **(B)** of kiwifruit [control (CK), H_2_S] with inoculation of *B*. *dothidea* during storage at 25°C. Vertical bars represented the standard errors. Letters indicate significant differences (*P* < 0.05).

The H_2_S treatment notably reduced lesion area expansion (*P* < 0.05) (Figure 1A). After 6 days of inoculation with *B. dothidea*, the lesion area of H_2_S-treated fruit was 38.41 mm^2^, which was 64.63% lower than the CK (108.58 mm^2^) (Figure 1B). The H_2_S treatment significantly reduced the lesion area and enhanced the induced disease resistance of kiwifruit after inoculation.

### Effects of H_2_S on the Growth of *B. dothidea*

**Figure 2:**
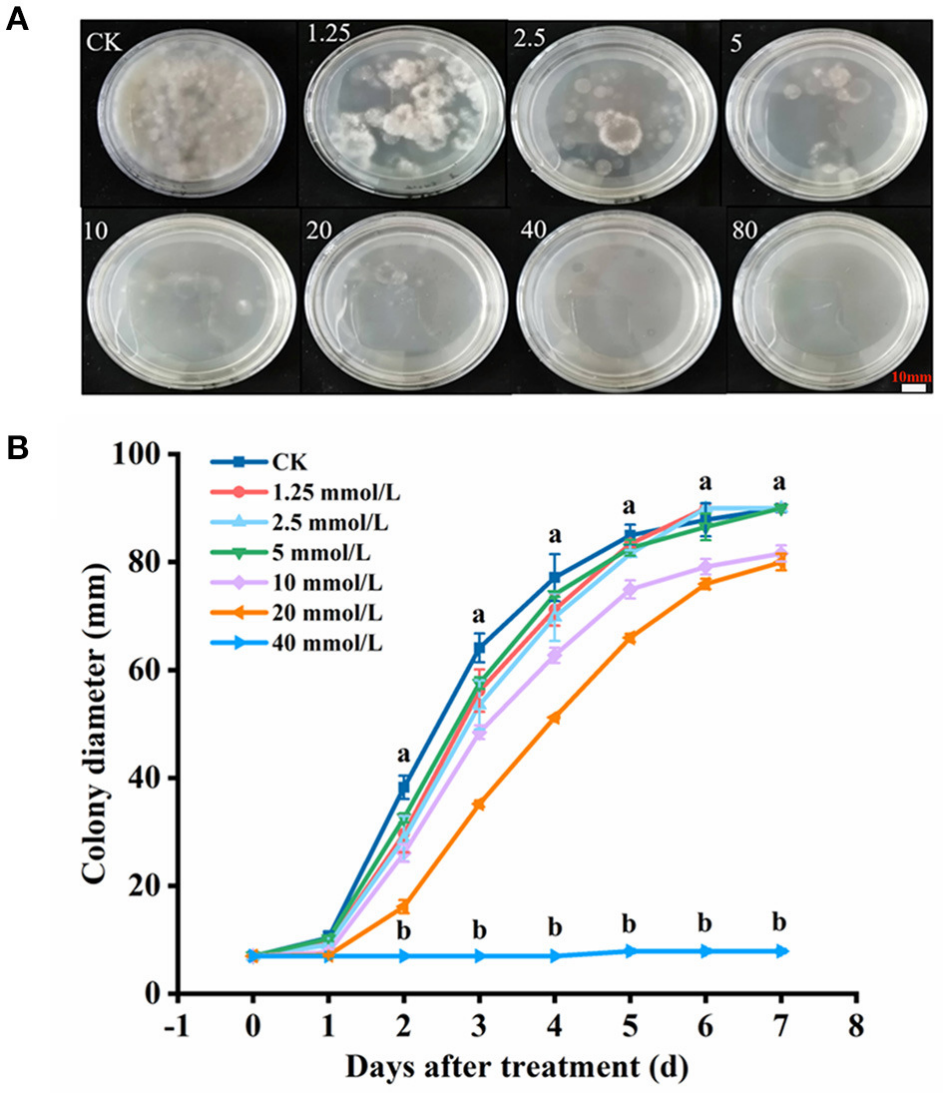
Mycelial growth **(A)** and colony diameter **(B)** of *B*. *dothidea* with H_2_S treatment. The NaHS solution (H_2_S donor) was added to make the final concentration of 0, 1.25, 2.5, 5, 10, 20, 40, and 80 mmol L^−1^, respectively. Vertical bars represent the standard errors. Letters indicate significant differences (*P* < 0.05).

The results indicated that H_2_S conspicuously restricted the mycelial growth of *B*. *dothidea* (*P* < 0.05) within 7 days of constant temperature culture, and MIC of NaHS was determined to be 20 mmol L^−1^ (Figure 2A). On the third day of culture, the colony diameter of MIC was 54.91% that of the CK, indicating that it possessed bacteriostatic properties. The mycelial growth of *B*. *dothidea* was entirely suppressed by 40 mmol L^−1^ (2MIC) NaHS (*P* < 0.05) (Figure 2B).

### Changes of Mycelial Weight, Extracellular Conductivity, Membrane Leakage, and MDA of *B. dothidea* After H_2_S Treatment

**Figure 3:**
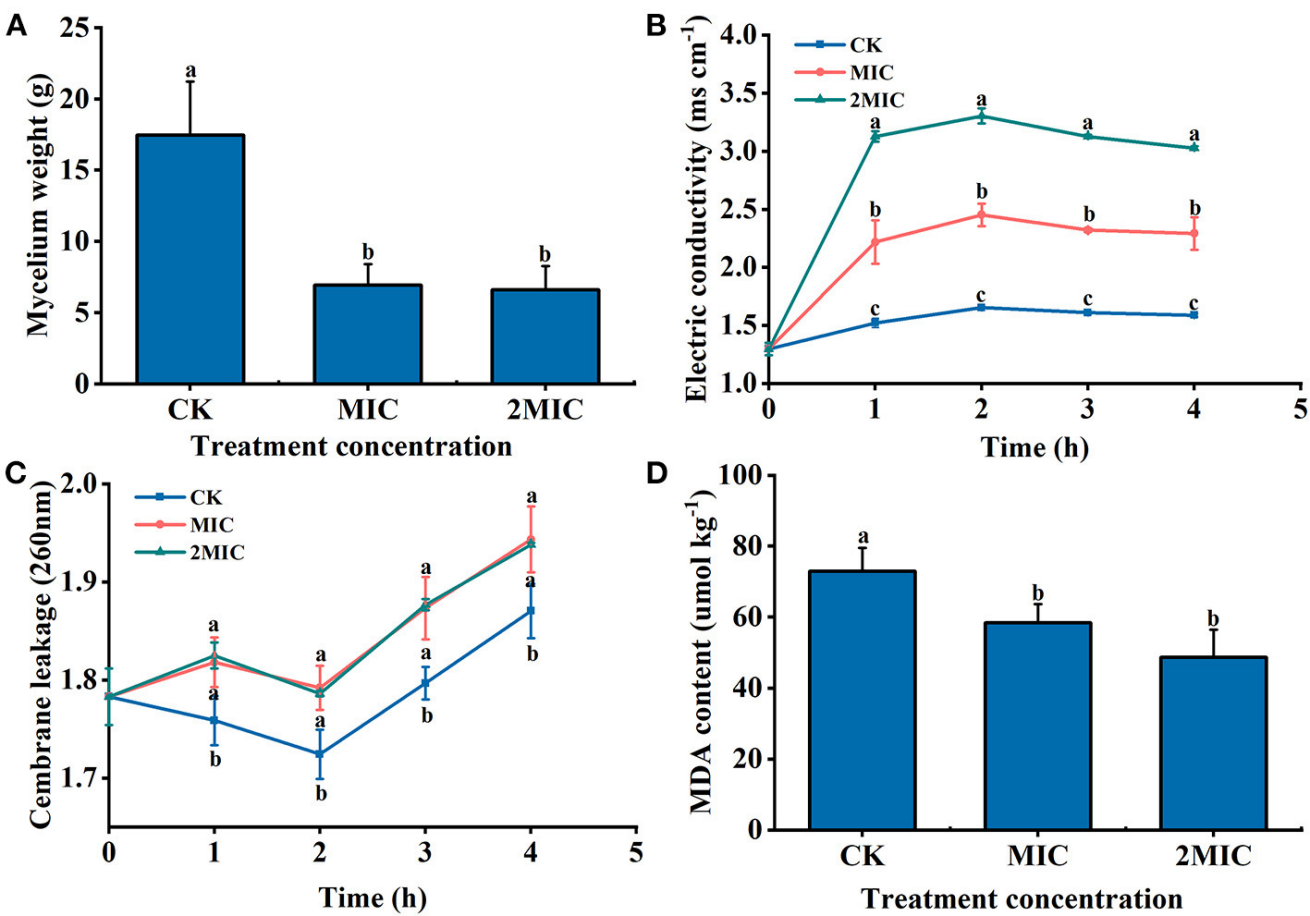
*B*. *dothidea* treated by NaHS [minimum inhibitory concentration (MIC), 2MIC]. **(A)** Mycelial weight; **(B)** extracellular conductivity; **(C)** the absorbance value of extracellular fluid at 260 nm; and **(D)** the content of malondialdehyde (MDA). Vertical bars represent the standard errors. Letters indicate significant differences (*P* < 0.05).

Compared with the CK, the mycelial weight of MIC and 2MIC treated with NaHS decreased by 60.27 and 62.12%, respectively (*P* < 0.05) (Figure 3A). Additionally, H_2_S treatment altered extracellular conductivity. The extracellular conductivity of MIC and 2MIC groups was 1.48 and 2 times higher than the CK at 2 h, respectively (*P* < 0.05) (Figure 3B). Additionally, it was found that the absorbance value of H_2_S-treated group decreased at first and then increased. The absorbance value of the treatment group at 260 nm was larger than the CK within 4 h (*P* < 0.05) (Figure 3C). Additionally, hyphae treated with H_2_S had a lower MDA content (*P* < 0.05). The MDA content in the CK group was 1.25 and 1.5 times larger than that in H_2_S treatment, respectively (Figure 3D).

### Changes of Defense-Selected Gene Expression After H_2_S Treatment

**Figure 4:**
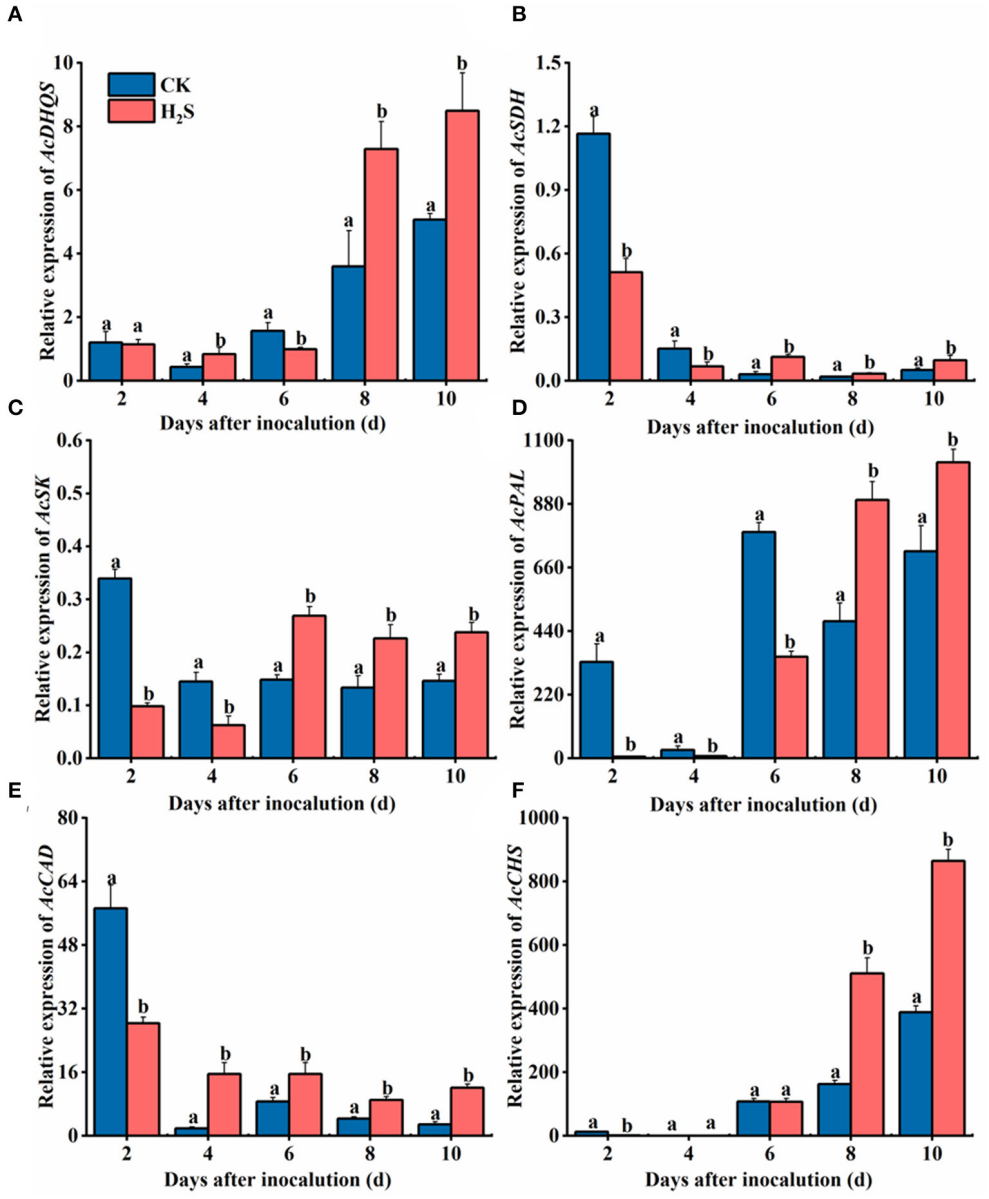
Expressions of *AcDHQS* **(A)**, *AcSDH* **(B)**, *AcSK* **(C)**, *AcPAL* **(D)**, *AcCAD* **(E)**, and *AcCHS* **(F)** genes in kiwifruit with inoculation of *B. dothidea* after the H_2_S treatment during storage at 25°C. Actin was used as an internal standard for each gene. Vertical bars represent the standard errors. Letters indicate significant differences (*P* < 0.05).

*AcDHQS* gene expression was conspicuously upregulated in H_2_S-treated kiwifruit samples on 8–10 days after inoculation. Specifically, the difference in gene expression was the largest on day 8, which was 2.03 times that of the CK (*P* < 0.05) (Figure 4A). On 6–10 days after inoculation, the expression of *AcSDH* and *AcSK* genes was upregulated in the presence of H_2_S (Figures 4B,C). The expression levels of *AcSDH* and *AcSK* genes in H_2_S-treated kiwifruit were elevated by 263.78 and 81.25% on day 6, respectively, compared with the CK. After 8 days of inoculation, *AcPAL* gene expression treated with H_2_S was markedly higher than the CK. Concretely, *AcPAL* gene expression in H_2_S-treated fruit was 1.89 times that of the CK on day 8 (*P* < 0.05) and reached the highest level on day 10 (Figure 4D). The gene expression level of *AcCAD* in H_2_S-treated fruit increased significantly during 4–10 days after inoculation (Figure 4E). However, *AcCAD* gene expression augmented during 8–10 days following inoculation (Figure 4F).

### Changes of Metabolites of Disease-Related Amino Acids After H_2_S Treatment

**Figure 5:**
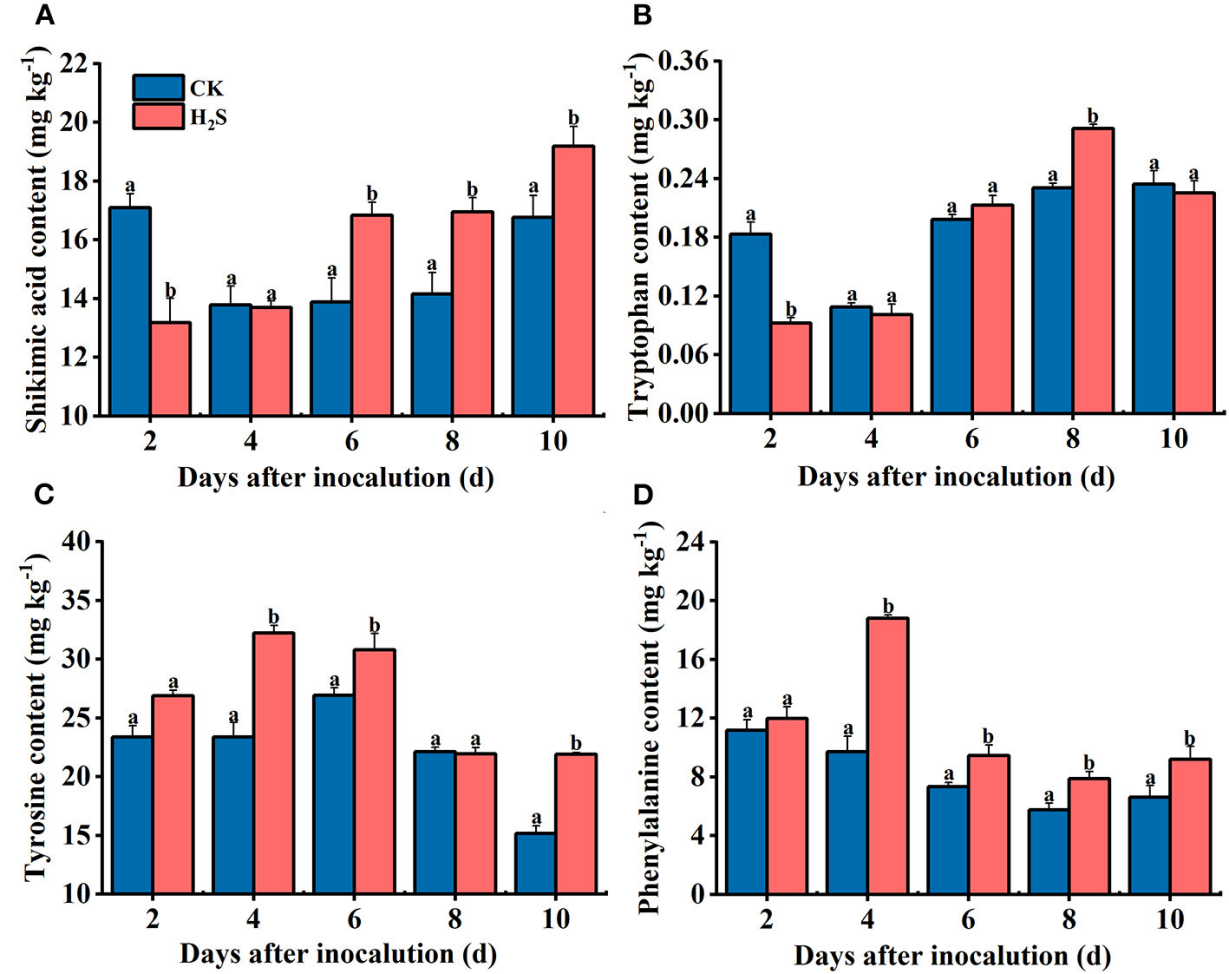
Shikimic acid **(A)**, tyrosine **(B)**, tryptophan **(C)**, and phenylalanine **(D)** contents in kiwifruit with inoculation of *B. dothidea* after the H_2_S treatment during storage at 25°C. Vertical bars represent the standard errors. Letters indicate significant differences (*P* < 0.05).

Shikimic acid is an intermediate metabolite of the shikimic acid pathway and an important precursor for aromatic amino acids. The shikimic acid content in H_2_S-treated fruit was higher than in CK during 6–10 days after inoculation, and the greatest difference was found on day 6, which was 21.24% higher than in CK fruit (*P* < 0.05) (Figure 5A). The tryptophan content in H_2_S-treated group increased gradually on 2–8 days after inoculation and then decreased after reaching a peak on day 8 (Figure 5B). Tyrosine and phenylalanine levels increased in H_2_S-treated fruit at 2–4 days and subsequently decreased on 4–10 days of storage (Figures 5C,D). On day 4 following inoculation, tyrosine and phenylalanine contents elevated by 27.46 and 48.43%, respectively, compared with the CK group (*P* < 0.05).

### Changes of Disease-Resistant Related Substances Content After H_2_S Treatment

**Figure 6:**
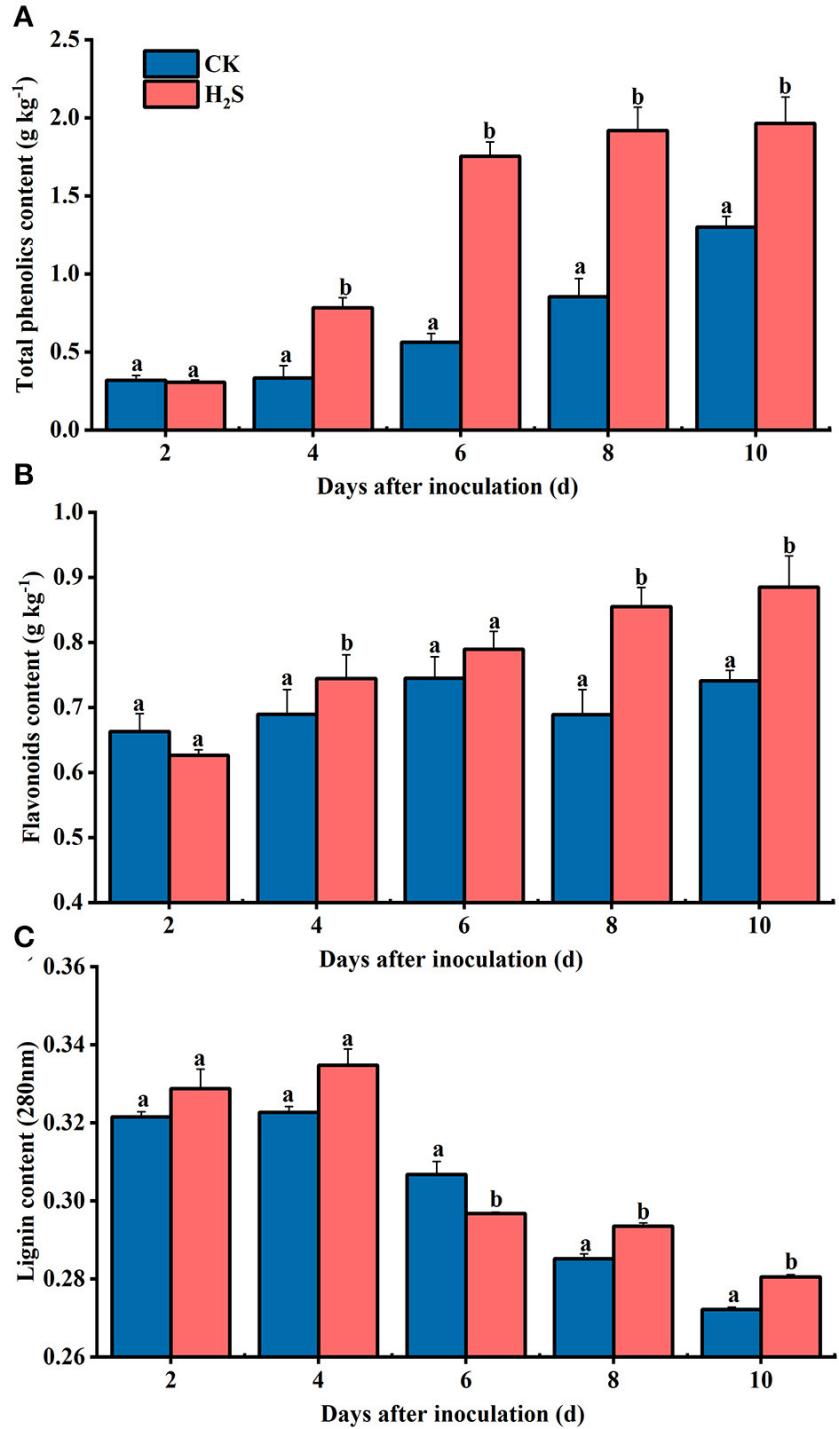
Contents of total phenolic **(A)**, flavonoids **(B)**, and lignin **(C)** in kiwifruit with inoculation of *B. dothidea* after H_2_S treatment during storage at 25°C. Vertical bars represent the standard errors. Letters indicate significant differences (*P* < 0.05).

In the whole storage period, the total phenol content in kiwifruit of the two groups increased steadily; the total phenol content in H_2_S-treated group from 4 to 10 days was about 131% higher than that of the CK on average (*P* < 0.05) (Figure 6A). The content of total flavonoids in CK and H_2_S-treated fruit exhibited a similar momentum to that of total phenol during storage (Figure 6B). The lignin content of CK fruit increased initially and then decreased during storage, reaching the maximum on day 4 (Figure 6C).

## Discussion

H_2_S is a plant signaling molecule that stimulates the natural defense mechanism. Studies have shown that the serum concentration of H_2_S in most structures of human body is 5.2 ×10^−6^ mol L^−1^, which can occur naturally in the human body, environment, and intestinal tract, and the enzymes existing in human body can be oxidized to produce harmless sulfate for detoxification (Kimura, 2011). In this research, the concentration of H_2_S fumigated fruits is very low, and the residual concentration is lower than the endogenous human plasma H_2_S level; therefore, H_2_S treatment will not adversely affect the safety of fruits. Numerous studies have revealed that H_2_S has a favorable effect on delaying maturation and senescence of fruit and vegetable and maintaining postharvest quality, yet, there are few studies on inducing disease resistance after harvest. Previous investigations manifested that H_2_S exhibits a good control effect on postharvest diseases such as sweet potatoes and pears, significantly reducing the lesion diameters and degree of damage (Hu et al., 2014; Tang et al., 2014). Furthermore, H_2_S effectively controlled the decay of apples, pears, tomatoes, kiwifruits, sweet oranges, and mandarins caused by *A. niger* and *P. italicum* (Fu et al., 2014). Our experiments indicated that the lesion area of kiwifruit was conspicuously restricted after H_2_S treatment. As a result, H_2_S treatment might induce disease resistance to control soft rot and quality deterioration of postharvest kiwifruit.

Although studies indicated that H_2_S seriously inhibits fungi propagation (i.e., *A. niger* and *P. italicum*), and H_2_S has a strong antifungal role on pathogens (Hu et al., 2014); the mechanism that mediates this response has not yet been reported. Our research manifested that H_2_S distinctly suppressed *B*. *dothidea* growth *in vitro*. The cell membrane has a paramount function in maintaining the content of sugars, protein, and inorganic salts required for cell life activities, and its injury can cause leakage of cell inclusions (Liu et al., 2019). Relative conductivity and membrane leakage are crucial indicators to measure the permeability of cell membranes, indicating the degree of cell membrane damage (Cheng et al., 2022). The results indicated that H_2_S increased the extracellular conductivity of *B. dothidea* and enlarged the degree of membrane leakage, thereby increasing the permeability of *B. dothidea* membrane and inhibiting pathogen growth. MDA is the pivotal product of membrane lipid peroxidation, and its content reflects the damage degree (Pan et al., 2019). MDA is a small water-soluble molecule that may be released into the culture medium outside the cell when the cell membrane is damaged. In our experiments, the MDA content in the hyphae was significantly reduced with H_2_S treatment. Thus, we proposed that H_2_S may affect lipid peroxidation of mycelial membrane of *B. dothidea*, change its permeability, and cause damage to cell membranes and organelles.

It is considered one of the most promising methods to control fruit diseases after harvest by inducing resistance to activate the defense system of the fruit itself, thereby strengthening disease resistance ability. As the basic component of protein, amino acids are critical in plants, particularly aromatic amino acids, which not only participate in various physiological processes but also meet the crucial functions of signal transmission and defense (Kumar et al., 2020). As a result, scholars should pay close attention to the plant disease resistance response due to the amino acid metabolism pathway. The phenylpropanoid metabolic pathway with phenylalanine as the starting substance has been extensively studied and has been shown to play a critical function in plant disease resistance. Phenylpropanoid metabolic pathway activation was catalyzed by phenylalanine ammonia lyase (PAL), and the outcomes of phenols, flavonoids, and lignin are typical antibacterial substances. Cinnamyl-alcohol dehydrogenase (CAD) catalyzes the conversion of cinnamaldehyde to cinnamyl alcohol, which is involved in lignin synthesis, whereas chalcone synthase (CHS) is mainly involved in flavonoid synthesis (Ge et al., 2018; Li et al., 2021). In previous research, the activity and gene expression level of PAL, CHS, and CAD in goji berry were activated, resulting in a higher content of phenylpropanoid metabolites, thus further enhancing disease resistance (Zhang et al., 2021a). In our study, the expression levels of *AcPAL, AcCAD*, and *AcCHS* in H_2_S-treated kiwifruit were markedly higher than in CK, but these expression levels were upregulated in the later stage. Additionally, H_2_S-treated fruit augmented the accumulation of total phenols, flavonoids, and lignin. The findings corroborated previous research on disease resistance of melatonin-treated litchi (Zhang et al., 2021b), nitric oxide-treated peach (Li et al., 2017), and kiwifruit (Yang et al., 2021).

In the entire amino acid metabolism pathway, in addition to the aforementioned phenylalanine, other amino acids (e.g., tyrosine and tryptophan) may jointly participate in inducing disease resistance in postharvest fruit (Wang et al., 2020a). As the upstream of the amino acid metabolism process, shikimic acid metabolism has an irreplaceable influence on enhancing plant resistance (Yokoyama et al., 2021). Shikimic acid pathway started with phosphoenolpyruvate (PEP) and erythritol-4-phosphate, which was catalyzed by 3-dehydroquinate synthase (DHQS), shikimate dehydrogenase (SDH), and shikimate kinase (SK) to produce aromatic amino acids, thus activating other pathways that help to enhance fruit resistance (Maeda and Dudareva, 2012). Our experimental results illustrated that H_2_S significantly upregulated the expression of *AcDHQS, AcSDH*, and *AcSK* genes in kiwifruit. Shikimic acid is also the precursor of gallic acid (GA) pathway (Awad et al., 2017). Tryptophan is one of the downstream metabolites in shikimic acid pathway. This study revealed that H_2_S treatment diminished tryptophan and shikimic acid contents in the early storage period and promoted their accumulation in fruit during the later stage of storage. This phenomenon may be because these two substances act as precursors of phenolic metabolic pathway such as alkaloids, providing sufficient substrates for subsequent reactions or may be related to GA pathway. In kiwifruit treated with H_2_S, endogenous tryptophan and tyrosine accumulation could be responsible for melatonin and dopamine biosynthesis and accumulation, respectively. Both molecules exhibit antioxidant activity and are crucial for attenuating fungal pathogens by signaling function. As an important precursor of phenylpropane metabolic pathway, the contents of tyrosine and phenylalanine in H_2_S-treated kiwifruit were significantly higher than those of CK during the storage time, consistent with the research of Ge et al. (2019b). Overall, these encouraging results demonstrated that H_2_S-induced disease resistance was linked to metabolites and gene expression levels associated with amino acid metabolism pathway in kiwifruit.

## Conclusion

The findings demonstrate that exogenous H_2_S exhibits a significant control effect on kiwifruit soft rot caused by *B. dothidea* and antifungal effect *in vitro*. H_2_S effectively increased the expression levels of *AcDHQS, AcSDH, AcSK, AcPAL, AcCAD*, and *AcCHS* genes, as well as the accumulation of some secondary metabolites involved in amino acid metabolism, including shikimic acid, tyrosine, tryptophan, phenylalanine, total phenol, flavonoids, and lignin. The molecular mechanism of amino acid metabolism in H_2_S-induced kiwifruit will be investigated using transcriptomic and metabolomic analyses in the future.

## Data Availability Statement

The original contributions presented in the study are included in the article/Supplementary Materials, further inquiries can be directed to the corresponding authors.

## Author Contributions

BD wrote the original draft and conducted the experiments. HD and WZ conducted visualization. JW and ZC conducted the investigation. YS, TH, and JY used software and resources. BD and ZG conducted formal analysis. JC and LZ conceptualized the research and did the supervision. LZ reviewed and edited the manuscript. All authors contributed to the article and approved the submitted version.

## Funding

This study was supported by the Natural Science Foundation of China (No. 32160733/31560219) and Science Foundation of Jiangxi Province (20212BAB205015).

## Conflict of Interest

The authors declare that the research was conducted in the absence of any commercial or financial relationships that could be construed as a potential conflict of interest.

## Publisher's Note

All claims expressed in this article are solely those of the authors and do not necessarily represent those of their affiliated organizations, or those of the publisher, the editors and the reviewers. Any product that may be evaluated in this article, or claim that may be made by its manufacturer, is not guaranteed or endorsed by the publisher.

## References

[B1] Awad M. A. Al-Qurashi A. D. Mohamed S. A. El-Shishtawy R. M. Ali M. A. (2017). Postharvest chitosan, gallic acid and chitosan gallate treatments effects on shelf life quality, antioxidant compounds, free radical scavenging capacity and enzymes activities of “Sukkari” bananas. J. Food Sci. Tech. 54, 447–457. 10.1007/s13197-016-2481-8 28242944 PMC5306039

[B2] Canfield C. A. Bradshaw P. C. (2019). Amino acids in the regulation of aging and aging-related diseases. Transl. Med. Aging 3, 70–89. 10.1016/j.tma.2019.09.001

[B3] Cheng X. M. Yang Y. Zhu X. R. Yuan P. Gong B. Y. Ding S. H.. (2022). Inhibitory mechanisms of cinnamic acid on the growth of *Geotrichum citri-aurantii*. Food Control 131:108459. 10.1016/j.foodcont.2021.108459

[B4] Dai Y. Wang Z. S. Leng J. S. Sui Y. Jiang M. G. Wisniewski M. (In Press). Eco-friendly management of postharvest fungal decays in kiwifruit. Crit. Rev. Food Sci. Nutri. 1–12. 10.1080/10408398.2021.1926908. 33998844

[B5] Dou Y. Chang C. M. Wang J. Cai Z. P. Zhang W. Du H. Y.. (2021). Hydrogen sulfide inhibits enzymatic browning of fresh-cut Chinese Water Chestnuts. Front. Nutr. 8:652984. 10.3389/fnut.2021.652984 34150826 PMC8212951

[B6] Fu L. H. Hu K. D. Hu L. Y. Li Y. H. Hu L. B. Yan H.. (2014). An antifungal role of hydrogen sulfide on the postharvest pathogens *Aspergillus niger* and *Penicillium italicum*. PLoS ONE 9:e104206. 10.1371/journal.pone.0104206 25101960 PMC4125178

[B7] Ge Y. H. Chen Y. R. Li C. Y. Zhao J. R. Wei M. L. Li X. H.. (2019a). Effect of sodium nitroprusside treatment on shikimate and phenylpropanoid pathways of apple fruit. Food Chem. 290, 263–269. 10.1016/j.foodchem.2019.04.010 31000046

[B8] Ge Y. H. Duan B. Li C. Y. Tang Q. Li X. Wei M. L.. (2018). γ-Aminobutyric acid delays senescence of blueberry fruit by regulation of reactive oxygen species metabolism and phenylpropanoid pathway. Sci. Hortic. 240, 303–309. 10.1016/j.scienta.2018.06.044

[B9] Ge Y. H. Li X. Li C. Y. Tang Q. Duan B. Cheng Y.. (2019b). Effect of sodium nitroprusside on antioxidative enzymes and the phenylpropanoid pathway in blueberry fruit. Food Chem. 295, 607–612. 10.1016/j.foodchem.2019.05.160 31174802

[B10] He J. L. Wu D. T. Zhang Q. Chen H. Li H. Y. Han Q. H.. (2018). Efficacy and mechanism of cinnamon essential oil on inhibition of *Colletotrichum acutatum* isolated from ‘Hongyang' Kiwifruit. Front. Microbiol. 9:1288. 10.3389/fmicb.2018.01288 29967599 PMC6015887

[B11] Hu K. D. Wang Q. Hu L. Y. Gao S. P. Wu J. Li Y. H.. (2014). Hydrogen sulfide prolongs postharvest storage of fresh-cut pears (*Pyrus pyrifolia*) by alleviation of oxidative damage and inhibition of fungal growth. PLoS ONE 9:e85524. 10.1371/journal.pone.0085524 24454881 PMC3893216

[B12] Jan R. Asaf S. Numan M. Kim K. M. (2021). Plant secondary metabolite biosynthesis and transcriptional regulation in response to biotic and abiotic stress conditions. Agronomy 11:968. 10.3390/agronomy1105096822047180

[B13] Jin L. Cai Y. Sun C. Huang Y. Yu T. (2019). Exogenous L-glutamate treatment could induce resistance against *Penicillium expansum* in pear fruit by activating defense-related proteins and amino acids metabolism. Postharvest Biol. Technol. 150, 148–157. 10.1016/j.postharvbio.2018.11.009

[B14] Kadotani N. Akagi A. Takatsuji H. Miwa T. Igarashi D. (2016). Exogenous proteinogenic amino acids induce systemic resistance in rice. BMC Plant Biol. 16:60. 10.1186/s12870-016-0748-x 26940322 PMC4778346

[B15] Kai K. Bi W. Sui Y. Hua C. Y. Liu Y. S. Zhang D. F. (2020). Curcumin inhibits Diaporthe phaseolorum and reduces postharvest decay in kiwifruit. Sci. Hortic. 259:108860. 10.1016/j.scienta.2019.108860

[B16] Kimura H. (2011). Hydrogen sulfide: its production and functions. Exp. Physiol. 96, 833–835. 10.1113/expphysiol.2011.057455 21527544

[B17] Kumar P. M. Maurer D. Feygenberg O. Ovadia A. Elad Y. Oren-Shamir M.. (2020). Phenylalanine: a promising inducer of fruit resistance to postharvest pathogens. Foods 9:646. 10.3390/foods9050646 32443417 PMC7278716

[B18] Li G. J. Zhu S. H. Wu W. X. Zhang C. Peng Y. Wang Q. G.. (2017). Exogenous nitric oxide induces disease resistance against *Monilinia fructicola* through activating the phenylpropanoid pathway in peach fruit. J. Sci. Food Agric. 97, 3030–3038. 10.1002/jsfa.8146 27859285

[B19] Li X. A. Li B. R. Min D. D. Ji N. N. Zhang X. H. Li F. J.. (2021). Transcriptomic analysis reveals key genes associated with the biosynthesis regulation of phenolics in fresh-cut pitaya fruit (*Hylocereus undatus*). Postharvest Biol. Technol. 181:111684. 10.1016/j.postharvbio.2021.111684

[B20] Lin X. C. Yang R. Dou Y. Zhang W. Du H. Y. Zhu L. Q.. (2020). Transcriptome analysis reveals delaying of the ripening and cell-wall degradation of kiwifruit by hydrogen sulfide. J. Sci. Food Agric. 100, 2280–2287. 10.1002/jsfa.10260 31944323

[B21] Liu R. L. Gao H. Y. Chen H. J. Fang X. J. Wu W. J. (2019). Synergistic effect of 1-methylcyclopropene and carvacrol on preservation of red pitaya (*Hylocereus polyrhizus*). Food Chem. 283, 588–595. 10.1016/j.foodchem.2019.01.066 30722915

[B22] Liu X. Y. Cui X. M. Ji D. C. Zhang Z. Q. Li B. Q. Xu Y.. (2021). Luteolin-induced activation of the phenylpropanoid metabolic pathway contributes to quality maintenance and disease resistance of sweet cherry. Food Chem. 342:128309. 10.1016/j.foodchem.2020.128309 33051099

[B23] Liu Y. Y. Ge Y. H. Bi Y. Li C. Y. Deng H. W. Dong B. Y. (2014). Effect of postharvest acibenzolar-S-methyl dipping on phenylpropanoid pathway metabolism in muskmelon (*Cucumis melo* L.) fruits. Sci. Hortic. 168, 113–119. 10.1016/j.scienta.2014.01.030

[B24] Maeda H. Dudareva N. (2012). The shikimate pathway and aromatic amino acid biosynthesis in plants. Annu. Rev. Plant Biol. 63, 73–105. 10.1146/annurev-arplant-042811-105439 22554242

[B25] Pan L. Y. Zhao X. Y. Chen M. Fu Y. Q. Xiang M. L. Chen J. Y. (2019). Effect of exogenous methyl jasmonate treatment on disease resistance of postharvest kiwifruit. Food Chem. 305:125483. 10.1016/j.foodchem.2019.125483 31610420

[B26] Prencipe S. Nari L. Vittone G. Gullino M. L. Spadaro D. (2016). Effect of bacterial canker caused by *Pseudomonas syringae* pv. *actinidiae* on postharvest quality and rots of kiwifruit ‘Hayward'. Postharvest Biol. Technol. 113, 119–124. 10.1016/j.postharvbio.2015.11.010

[B27] Rajestary R. Landi L. Romanazzi G. (2021). Chitosan and postharvest decay of fresh fruit: meta-analysis of disease control and antimicrobial and eliciting activities. Compr. Rev. Food Sci. Food Saf. 20, 563–582. 10.1111/1541-4337.12672 33443789

[B28] Sun C. Jin L. F. Cai Y. T. Huang Y. N. Zheng X. D. Yu T. (2019). L-Glutamate treatment enhances disease resistance of tomato fruit by inducing the expression of glutamate receptors and the accumulation of amino acids. Food Chem. 293, 263–270. 10.1016/j.foodchem.2019.04.113 31151610

[B29] Sun Y. Zhang W. Zeng T. Nie Q. X. Zhang F. Y. Zhu L. Q. (2015). Hydrogen sulfide inhibits enzymatic browning of fresh-cut lotus root slices by regulating phenolic metabolism. Food Chem. 177, 376–381. 10.1016/j.foodchem.2015.01.065 25660900

[B30] Tang J. Hu K. D. Hu L. Y. Li Y. H. Liu Y. S. Zhang H. (2014). Hydrogen sulfide acts as a fungicide to alleviate senescence and decay in fresh-cut sweetpotato. HortScience 49, 938–943. 10.21273/HORTSCI.49.7.938

[B31] Wang L. Luo Z. S. Yang M. Y. Li D. Qi M. Xu Y. Q.. (2020a). Role of exogenous melatonin in table grapes: first evidence on contribution to the phenolics-oriented response. Food Chem. 329:127155. 10.1016/j.foodchem.2020.127155 32512393

[B32] Wang S. P. Zhou Y. H. Luo W. Deng L. L. Yao S. X. Zeng K. F. (2020b). Primary metabolites analysis of induced citrus fruit disease resistance upon treatment with oligochitosan, salicylic acid and Pichia membranaefaciens. Biol. Control 148:104289. 10.1016/j.biocontrol.2020.104289

[B33] Yang R. Du H. Y. Sun Y. Zhang F. Y. Zhang W. Wan C. P.. (2021). Effects of nitric oxide on the alleviation of postharvest disease induced by *Penicillium italicum* in navel orange fruits. Int. J. Food Sci. Tech. 56, 5259–5267. 10.1111/ijfs.15054

[B34] Yokoyama R. De Oliveira M. V. Kleven B. Maeda H. A. (2021). The entry reaction of the plant shikimate pathway is subjected to highly complex metabolite-mediated regulation. Plant Cell 33, 671–696. 10.1093/plcell/koaa042 33955484 PMC8136874

[B35] Yun Z. Zhu F. Liu P. Zeng Y. L. Xu J. Cheng Y. J.. (2015). Sweating treatment enhances citrus fruit disease resistance by inducing the accumulation of amino acids and salicylic acid-induced resistance pathway. Physiol. Plantarum 155, 109–125. 10.1111/ppl.12340 25893482

[B36] Zeier J. (2013). New insights into the regulation of plant immunity by amino acid metabolic pathways. Plant Cell Environ. 36, 2085–2103. 10.1111/pce.12122 23611692

[B37] Zhang C. Long Y. H. Li J. H. Li M. Xing D. K. An H. M.. (2020). A chitosan composite film sprayed before pathogen infection effectively controls postharvest soft rot in kiwifruit. Agronomy 10:265. 10.3390/agronomy10020265

[B38] Zhang H. Y. Liu F. R. Wang J. J. Yang Q. R. Wang P. Zhao H. J.. (2021a). Salicylic acid inhibits the postharvest decay of goji berry (*Lycium barbarum* L.) by modulating the antioxidant system and phenylpropanoid metabolites. Postharvest Biol. Technol. 178:111558. 10.1016/j.postharvbio.2021.111558

[B39] Zhang Z. K. Wang T. Liu G. S. Hu M. J. Yun Z. Duan X. W.. (2021b). Inhibition of downy blight and enhancement of resistance in litchi fruit by postharvest application of melatonin. Food Chem. 347:129009. 10.1016/j.foodchem.2021.129009 33444889

[B40] Zhu L. Q. Wang W. Shi J. Y. Zhang W. Shen Y. G. Du H. Y.. (2014). Hydrogen sulfide extends the postharvest life and enhances antioxidant activity of kiwifruit during storage. J. Sci. Food Agric. 94, 2699–2704. 10.1002/jsfa.6613 25328925

[B41] Zhu Y. Y. Yu J. Brecht J. K. Jiang T. J. Zheng X. L. (2016). Pre-harvest application of oxalic acid increases quality and resistance to *Penicillium expansum* in kiwifruit during postharvest storage. Food Chem. 190, 537–543. 10.1016/j.foodchem.2015.06.001 26213007

