# Supplementary material for: An Antifungal Role of Hydrogen Sulfide on Botryosphaeria Dothidea and Amino Acid Metabolism Involved in Disease Resistance Induced in Postharvest Kiwifruit
Source: Front Plant Sci. 2022 Jun 16;13:888647. doi: 10.3389/fpls.2022.888647 (PMC9244146; doi:10.3389/fpls.2022.888647)
Supplement: Supplementary file 1 [file Table_1.docx]

**Table S1.** Sequences of primers used in quantitative real-time RT-qPCR

| **Gene ID** | **Gene name** | **Primer sequence 5’→3’** | **Product size (bp)** |
| --- | --- | --- | --- |
| Ach05g207581 | DHQS | F: GGTCGAACGCGATTACTGAG | 156 |
|  |  | R: ACCATGAACATGCCTCTGGA |  |
| Ach28g456111 | SDH | F: TTCCGGCCTGAGAATGGAAT | 200 |
|  |  | R: ACTTACCACGATTGCTCCCA |  |
| Ach04g434771 | SK | F: CGATGCACCGTGATGCTAAT | 103 |
|  |  | R: TGGGACAAGTTCAAGAGGCA |  |
| Ach08g066341 | PAL | F: TGCTCGTACGGATCAACACT | 165 |
|  |  | R: TAAGGGCAAGCATGGAGTGA |  |
| Ach07g224201 | CAD | F: ACAGTCTGCCTCTTGATGCT | 139 |
|  |  | R: TGGCAAACTTAACCGCTACG |  |
| Ach08g238171 | CHS | F: CAAGCGCCTCATGATGTACC | 179 |
|  |  | R: CCCATCACCGAATAGAGCCT |  |
| Reference gene | Actin | F: TGGAATGGAAGCTGCAGGA | 104 |
|  |  | R: CACCACTGAGCACAATGTTGC |  |

Target genes and Actin were identified from a kiwifruit genome database (<http://kiwifruitgenome.org/home>).
